# Virtual Care for Patients with Advanced Well Differentiated Gastroenteropancreatic Neuroendocrine Tumor (GEP-NET)

**DOI:** 10.3390/curroncol31020071

**Published:** 2024-02-08

**Authors:** William J. Phillips, Michelle Pradier, Rachel Goodwin, Michael Vickers, Tim Asmis

**Affiliations:** 1 Division of Medical Oncology, The University of Ottawa, Ottawa, ON K1H 8L6, Canada; 2 Faculty of Medicine, The University of Ottawa, Ottawa, ON K1H 8L6, Canada; 3 Division of Medical Oncology, The University of Ottawa, The Ottawa Hospital Cancer Centre, 501 Smyth Rd, Ottawa, ON K1H 8L6, Canada

**Keywords:** virtual care, neuroendocrine tumor, carcinoid, oncology, COVID-19

## Abstract

Introduction: The COVID-19 pandemic resulted in an unprecedent shift towards virtual cancer care, including the care of gastroenteropancreatic neuroendocrine tumors (GEP-NETs). The aim of this study was to evaluate the use of virtual care for GEP-NETs during the COVID-19 pandemic at a high-volume academic cancer center. Methods: This retrospective, observational study performed at the Ottawa Hospital Cancer Center in Canada evaluated adult patients with GEP-NETs seen in consultation by medical oncology between 1 June 2019 and 31 December 2022. Demographic, clinicopathologic, cancer treatment and visit data were collected. Univariable and multivariable analyses assessed the relationship between patient characteristics and virtual care use. Results: A total of 103 patients with well-differentiated GEP-NETS were included. Overall, 18/103 (17.5%) consults and 594/781 (76.1%) follow-ups were performed virtually. All consultation visits returned to in-person assessment by 2022, while 67.0% and 41.4% follow-ups remained virtual in 2022 and 2023, respectively. The year of follow-up, sex, employment and Charlston comorbidity index were associated with virtual follow-up use in the multivariable analysis. Discussion: Virtual care remained a predominant method of GEP-NET patient assessment in the peri-pandemic period. These results highlight an opportunity to improve access to subspecialty neuroendocrine cancer care through the continued use of virtual care.

## 1. Introduction

Neuroendocrine tumors (NETs) are a group of cancers arising from neuroendocrine cells. Gastroenteropancreatic neuroendocrine tumors (GEP-NETs) arise from the gastrointestinal organs, including the pancreas, and make up approximately 70% of all NETs [1,2,3]. Although the incidence of GEP-NETs is low (2–5/100,000 people), their prevalence is significantly higher (35/100,000 people) [4]. Furthermore, the prevalence of GEP-NETs has steadily increased in recent years. In the United States, it is estimated that GEP-NETs have increased six-fold in the last four decades [5]. Large data registries also suggest a significant increase in the incidence of GEP-NETs in Canada [6]. This has largely been attributed to the routine use of endoscopy for colorectal cancer screening and the increasing availability of abdominal imaging. As a result, the volume of GEP-NET patients being assessed and followed by medical oncology has never been higher.

When the coronavirus 2019 (COVID-19) pandemic was declared, there was a rapid shift towards virtual care to prevent its spread. This transition occurred over a matter of days. It was estimated that the largest Canadian academic cancer centers performed approximately 514 visits per day [7]. This contrasts significantly with pre-pandemic cancer care, when nearly all oncology follow-up was performed through in-person assessment.

Virtual care is broadly defined as a method of patient assessment that is performed over the telephone or video-conferencing software. The concept of virtual care is not new and has been examined in academia for many decades, but it was only utilized sparsely in clinical practice up until the pandemic [8]. The pandemic provided proof that virtual cancer care was feasible on a population level. Throughout the pandemic, health policy was dynamic and precautions depended on the current prevalence of COVID-19 within one’s community. These policies often guided the decision of whether to follow up with patients in person versus virtually. As time passed, the COVID-19 restrictions reduced as less severe variants were becoming more common and vaccines were deployed at a large scale [9]. On 5 May 2023, the World Health Organization (WHO) declared the COVID-19 pandemic public health emergency over [10]. The infrastructure for virtual care still exists despite the pandemic being declared over, providing flexibility to patients and clinicians to decide on their preferred method of assessment.

We chose to study virtual care use in GEP-NET patients, as GEP-NETs exhibit several features that make them a good fit for virtual care. Firstly, this is a rare disease that requires specialty neuroendocrine care, which may be geographically distant from the patient’s primary residence. Virtual care can provide improved access and convenience for follow-up. Further, well-differentiated GEP-NETs that present with advance disease often progress slowly and may be managed for many years with active surveillance or somatostatin analogues (SSAs), depending on their underlying biology. Virtual care is thought to be well suited for patients undergoing a stable treatment plan.

In this study, we sought to evaluate the real-world use of virtual care for GEP-NET patients followed by medical oncology at a large Canadian academic cancer center. We believe that these data provide insights into how virtual care use has changed over the pandemic, specifically in the later stages of the pandemic period, when both in-person and virtual assessments were available.

## 2. Materials and Methods

This study received approval from the Ottawa Hospital Research Institute’s local research ethics board (REB).

### 2.1. Study Population and Design

This retrospective cohort study was performed at the Ottawa Hospital Cancer Centre. Patients who were 18 years or older, diagnosed with GEP-NETs and seen by medical oncology for an initial consultation between 1 June 2018 and 31 December 2022 were included. High-grade GEP-NETs, defined as Ki67 > 20% and neuroendocrine carcinomas (NECs) were excluded, as they carry a more aggressive underlying biology, making them less suited for virtual care, which was the primary focus of this study. Patients diagnosed with another metastatic (stage 4) malignancy requiring systemic therapy were also excluded.

The study’s index date was defined as the time of initial consultation with medical oncology. Baseline characteristics were collected until this date. Follow-up data were gathered until 31 January 2023 based on the lack of availability of administrative data at the time of analysis. 

### 2.2. Patient Characteristics

Patient information was gathered from the Ottawa Hospital Cancer Centre’s electronic medical records (EMR). Baseline demographic and clinical characteristics included age at initial consultation, sex, employment status, medical oncology provider (anonymized) and distance to cancer center, which was calculated as the distance required to drive from the patient’s permanent address to the closest Ottawa Hospital Cancer Centre site. Baseline health comorbidities were estimated using the Charlston comorbidity index, which was modified to exclude points allocated from a cancer diagnosis. Cancer characteristics collected included the year of diagnosis, stage, primary tumor site, presence of metastasis, sites of metastasis and local therapy (debulking surgery, radiation therapy). Information on histology, grade and Ki-67 was gathered from pathology reports. Systemic therapies were categorized based on the type of treatment received. These included somatostatin analogues (SSAs), targeted therapies, peptide receptor radionuclide therapy (PRRT) and cytotoxic chemotherapy. All lines of systemic therapy were counted.

### 2.3. Outcomes of Interest

Visit information was collected from hospital administrative data coding the type of visit performed. The primary outcome was the use of virtual care. Virtual visits consisted of telephone encounters or electronic video conferencing. Visits were categorized into consultation and follow-up assessments. Consultation visits represented the initial encounter with medical oncology, while follow-ups comprised all subsequent encounters. Consultations were not collected after 31 December 2022, while follow-ups were collected until the end of the follow-up period.

### 2.4. Statistical Analysis

Descriptive statistics were used to describe the study’s baseline characteristics. Binary and ordinal variables were reported as a frequency (n) and percentage (%). Continuous variables were recorded as ranges, or as the median and standard deviation (SD). The visit type was evaluated by the year of the visit and stratified by consultation versus follow-up. Binary logistic regression was used to assess the relationship between patient characteristics and virtual care follow-up use. Both univariable and multivariable analyses were performed. Multivariable analysis included the following variables: year, stage, age, sex, Charlston comorbidity index, employment, distance to cancer center, location, Ki67, receipt of any systemic therapy and provider. Two-sided 95% confidence intervals were reported in all regression analyses. Statistical significance was set to *p* ≤ 0.05. Statistical analysis was performed on SPSS (IBM Corp. Released 2021. IBM SPSS Statistics for Windows, Version 28.0. IBM Corp, Armonk, NY, USA). Figures were created using Microsoft Excel (Microsoft Corp. Released 2021. Microsoft Excel for Mac, Version 16.74, Redmond, WA, USA).

## 3. Results

### 3.1. Sample Characteristics

There were 103 patients included in the study. Baseline patient characteristics are summarized in Table 1. The median age was 60.5 years (SD = 13.6) and 49 (47.1%) patients were female. There were 19 (18.3%) patients seen in consultation in 2019, 27 (26.0%) patients in 2020, 38 (36.5%) patients in 2021 and 20 (19.2%) patients in 2022. The most common location of the primary tumor was the small bowel (n = 57, 55.3%), followed by the pancreas (n = 22, 21.4%) and colon (n = 14, 13.6%). There were 50 (48.1%) patients with de novo metastatic disease at presentation. Sites of metastases included the liver (n = 43, 41.7%), intra-abdominal lymph nodes (n = 18, 17.5%), bone (n = 12, 11.7%) and peritoneal deposits (n = 9, 8.7%). There were 68 (66.0%) patients who underwent prior radical surgery, while only five patients (4.9%) underwent debulking surgery and three (2.9%) received radiotherapy. Systemic therapy was received by 50 (48.1%) patients. The most common systemic therapy was SSA (n = 48, 46.2%), while six (5.8%) patients received chemotherapy, two (1.9%) PRRT and one (1.0%) targeted therapy.

### 3.2. Virtual Care Use by Year

**Figure 1:**
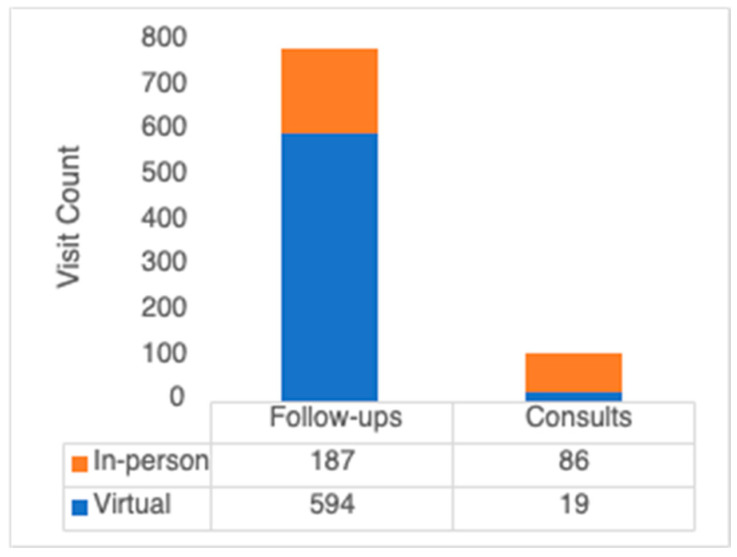
The distribution of virtual care use stratified by consultation and follow-up visits through the study period.

**Figure 2:**
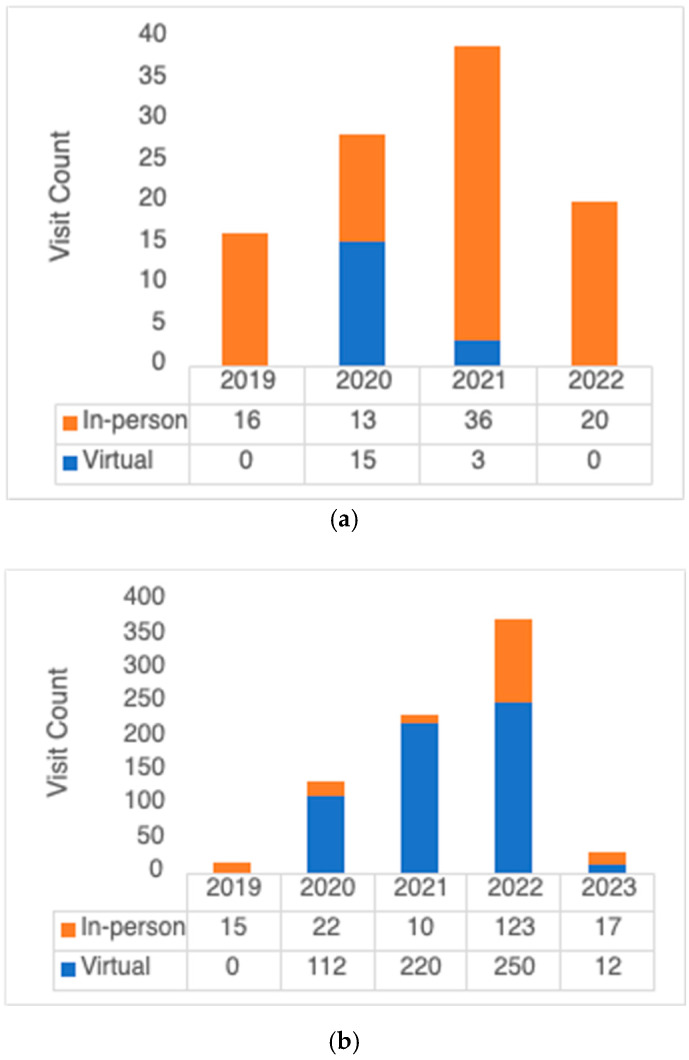
Virtual care use by year of appointment: (**a**) consultations, (**b**) follow-up visits.

During the study period, there were 103 consultation and 781 follow-up visits. The proportions of in-person and virtual encounters are depicted in Figure 1. Of the 103 consultations, 18 (17.5%) were virtual. In 2020, the proportion of virtual consultations was the highest, at 53.6%. There were no virtual consultations in 2022. Of the 781 follow-up visits, 594 (76.1%) were virtual. The proportion of virtual follow-ups was highest in 2020 and 2021, at 83.6% and 95.6%, respectively. The proportion of virtual visits declined to 67.0% in 2022, followed by 41.4% in 2023. The use of virtual care by year is shown in Figure 2.

### 3.3. The Relationship between Patient Characteristics and Virtual Care Use

The relationship between baseline characteristics and virtual care follow-up use was assessed using binary logistic regression modeling. Consultation visits were not assessed, as no consultation visits were performed virtually by 2022. Univariable analysis identified that follow-up in 2021 (OR = 4.32, CI = 1.98–9.44), 2022 (OR = 0.39, CI = 0.24–0.66) and 2023 (OR = 0.14, CI = 0.058–0.33) and being retired (OR = 0.57, CI = 0.41–0.080) were associated virtual care use; see Table 2. In the multivariable analysis, factors associated with virtual care use included follow-up in 2021 (OR = 4.67, CI = 2.03–10.73), 2022 (OR = 0.40, CI = 0.23–0.069) or 2023 (OR = 0.14, CI = 0.055–0.37), a higher Charlston Comorbidity index (OR = 1.88, CI = 1.03–3.43), being female (OR = 0.63, CI = 0.40–0.98) and retirement (OR = 0.25, CI = 0.13–0.45); see Table 3. Age, stage, distance to the cancer center, location of the primary tumor, Ki67, active systemic therapy and the medical oncology provider were not associated with virtual care use.

## 4. Discussion

The COVID-19 pandemic resulted in a rapid, unprecedent shift towards virtual care and proved that the widespread adoption of virtual oncology care was feasible. This study shows that virtual care remains a predominant method of follow-up for patients with GEP-NETs, even with the returned availability of in-person assessments. This contrasts with consultation visits, which had largely returned to in-person assessment by 2022. This suggests that virtual follow-up may be preferred by patients and/or clinicians and highlights an opportunity to improve the accessibility of specialized NET care through the ongoing use of virtual assessments. 

In general, well-differentiated GEP-NETs carry a different biology and natural history compared to other gastrointestinal adenocarcinomas, with a median overall survival of 9.3 years [11]. Local therapy, such as surgery or radiotherapy, is indicated upfront in patients with localized or oligo-metastatic disease [12]. Most patients with unresectable advanced disease can initiate first-line SSA therapy and avoid more intensive treatments such as chemotherapy, targeted agents or PRRT until progression [13,14]. Although virtual care has been shown to be safe in patients undergoing active treatment and even combination intravenous chemotherapies, its role is felt to be greatest in patients with a stable clinical status [15,16,17]. GEP-NETs may be well suited for virtual care under this principle. In our cohort, approximately 50% of patients were treated with systemic therapy and the majority received SSAs. It is worth noting that our study population represented a cohort of lower-risk patients. The majority had a Ki67 < 3% and only approximately half had advanced disease at diagnosis. We also excluded high-grade GEP-NETs and NECs. These tumors have a more aggressive natural history and may not be ideally suited for virtual care. 

It is interesting that, later in the pandemic, consultations were exclusively performed in person, while a significant number of follow-up visits were performed virtually. This likely reflects the importance of establishing a rapport face-to-face with patients initially and performing a baseline physical examination. The subsequent use of virtual follow-up may be related to its increased convenience and reduced travel-related expenses for patients. It may also contribute to normalizing the patient’s experience of cancer care by minimizing interruptions to their daily routine for follow-up. 

In the peri-pandemic period, the year of follow-up remains the strongest predictor of virtual care use by multivariable analysis. This is almost certainly explained by COVID-19 public health precautions. At the start of the pandemic, virtual care was universally adopted to prevent the spread of COVID-19. This period corresponded to the highest use of virtual care. As public health restrictions gradually eased, virtual care use decreased but still accounted for a significant number of encounters. By January 2023, 41.4% of follow-up encounters remained virtual. Being retired was associated with virtual care use, which may reflect the importance of work interruptions, potentially resulting in lost productivity or income, to patients who are employed. A higher Charlston comorbidity index, which is a marker of the general health status, was associated with increased virtual care use. We suspect this is multifactorial but may be related to reducing an already high number of medical appointments and the time spent in hospital. Females were less likely to receive virtual care. This trend is unclear, but it is well established that there are gender differences in the utilization of health services [18]. To our surprise, patients were equally likely to receive virtual care regardless of the geographic distance from the cancer center. This may reflect a shift in attitudes towards individuals’ time management irrespective of whether a patient resides in a rural or urban community. There were no differences in virtual care use between different providers or between patients receiving active systemic therapy. 

Virtual care has several advantages for patients with well-differentiated GEP-NETs. It offers increased comfort and convenience. This is supported by patient-reported outcomes showing a high level of satisfaction with virtual oncology care [19,20]. It also reduces patients’ travel time and associated costs. This is relevant in the context of GEP-NETs, as patients often receive oncology care at a specialized NET treatment center, which may be significantly further than their closest cancer center [21]. Virtual care provides flexibility to patients and can reduce the time away from work, family and other commitments. It also seems to be safe, even in patients undergoing active treatment for cancer, although high-quality data are lacking [16]. From a system standpoint, virtual care is efficient and cost-effective [7,22]. This is an important consideration, as oncology care is facing substantial economic and resource pressures due to increasing patient volumes. Virtual care may also help to facilitate improved access to clinical trials, as it is now an acceptable means of ongoing patient care. For this to occur, research protocols will need to be creative in offering a component of virtual care and ethics boards need to recognize the importance of allowing virtual care to improve accrual. 

There is still uncertainty around the routine use of virtual care in clinical practice. Specifically, patient selection for virtual care remains unclear. The decision to use virtual care is typically left at the discretion of individual physicians. Virtual care may not be the best modality for serious illness discussion or bad news disclosure [23]. It may also be more challenging to establish a patient rapport using virtual care, which may explain why consultation visits are usually performed in person. Other concerns include access to and an understanding of video-conferencing technology and healthcare worker renumeration, as well as patient privacy [24]. With these points in mind, further work is required to standardize virtual care as an option for patients with GEP-NETs.

There are limitations in this study. The Ottawa Hospital Cancer Centre is a high-volume, tertiary-care neuroendocrine treatment center with a catchment area of 1.4 million people. Despite this large referral base, this is a single-center study and may lack applicability to other institutions. Missing data were minimal, but inconclusive or non-diagnostic pathology reports did impact the full reporting of Ki67 and mitotic rates. Our study focused on the care of well-differentiated GEP-NETs early in their disease course, so its findings may not be representative of patients with high-grade tumors or later in their disease course.

## 5. Conclusions

In this cohort of patients with well-differentiated GEP-NETs followed by medical oncology at a neuroendocrine specialty center, virtual follow-up was the predominant method of patient assessment. This was true even later in the COVID-19 pandemic, when the availability of in-person assessments returned. Comorbidities, sex and employment were identified as factors associated with virtual care use. Overall, our data suggest that virtual care remains an important method of patient assessment and highlight an opportunity to improve access to subspecialty neuroendocrine cancer care.

## Figures and Tables

**Table 1:** Patient baseline demographic, clinical and cancer characteristics represented as frequency (n) and percentage (%).

Characteristic	Description	Frequency	Percentage
Age	<50	19	18.5%
	50–59	29	28.2%
	60–69	34	33.0%
	70–79	17	16.5%
	80+	4	3.9%
Sex	Female	49	47.1%
Charlston score (modified)	0–1	36	34.6%
	2–4	56	53.8%
	5+	12	11.5%
Employment	Employed	55	56.1%
	Retired	41	41.8%
	Disability	2	2.0%
Distance to cancer center	<20	58	55.8%
	20–49	22	21.2%
	50+	24	23.1%
Provider	Provider A	35	33.7%
	Provider B	46	44.2%
	Provider C	23	22.1%
Primary location	Small Bowel	57	55.3%
	Colon	14	13.6%
	Pancreas	22	21.4%
	Other/Unknown	10	9.7%
De novo metastatic disease	-	50	48.1%
Ki67	<3	63	67.7%
	3–20	30	32.3%
Year of oncology consultation	2019.0	19	18.3%
	2020.00	27	26.0%
	2021.00	38	36.5%
	2022.00	20	19.2%
Radical surgery	-	68	66.0%
Debulking surgery	-	5	4.9%
Radiation to metastatic site	-	3	2.9%
Any systemic therapy	-	50	48.1%
Somatostatin analogue	-	48	46.2%
Chemotherapy	-	6	5.8%
Targeted therapy	-	1	1.0%
Peptide receptor radionucleotide therapy	-	2	1.9%

**Table 2:** Univariable analysis of the relationship between patient characteristics and virtual follow-up use by binary logistic regression, reported with odds ratio and two-sided 95% confidence interval.

Characteristic	Description	OR (95% CI)	*p*-Value
Year of follow-up appointment	2020 (comparison)	-	-
	2019	-	-
	2021	4.32 (1.98–9.44)	<0.001
	2022	0.39 (0.24–0.66)	<0.001
	2023	0.14 (0.058–0.33)	<0.001
De novo metastases	-	0.54 (0.63–1.27)	0.89
Sex	Female	0.80 (0.57–1.11)	0.18
Age	-	1.06 (0.91–1.25)	0.45
Employment	Working (comparison)	-	-
	Retired	0.57 (0.41–0.80)	0.001
	Disability	2.96 (0.68–12.81)	0.15
Charlston score (modified)	-	1.28 (0.96–1.70)	0.091
Distance to cancer center	-	1.05 (0.86–1.28)	0.65
Location	Small bowel (comparison)	-	-
	Pancreas	0.80 (0.43–1.49)	0.49
	Colon	1.45 (0.95–2.21)	0.082
	Unknown/other	0.66 (0.31–1.40)	0.28
Ki67	>3%	1.21 (0.94–1.56)	0.14
Active systemic therapy	-	0.76 (0.53–1.09)	0.13
Provider	A (comparison)	-	-
	B	1.10 (0.76–1.61)	0.61
	C	0.98 (0.63–1.54)	0.94

**Table 3:** Multivariable analysis of the relationship between patient characteristics and virtual follow-up use by binary logistic regression, reported with odds ratio and two-sided 95% confidence interval.

Characteristic	Description	OR (95% CI)	*p*-Value
Year of follow-up appointment	2019	-	-
	2020 (comparison)	-	-
	2021	4.67 (2.03–10.73)	<0.001
	2022	0.40 (0.23–0.69)	<0.001
	2023	0.14 (0.055–0.37)	<0.001
De novo metastases	-	0.76 (0.36–1.60)	0.47
Sex	Female	0.63 (0.40–0.98)	0.04
Age	-	1.29 (0.90–1.84)	0.16
Employment	Working (comparison)	-	-
	Retired	0.25 (0.13–0.45)	0.040
	Disability	1.58 (0.26–9.43)	0.62
Charlston score	-	1.88 (1.03–3.43)	0.039
Distance to cancer center (km)	-	1.14 (0.87–1.49)	0.34
Location	Small bowel (comparison)	-	-
	Pancreas	0.68 (0.30–1.54)	0.36
	Colon	1.29 (0.75–2.21)	0.36
	Unknown/other	0.71(0.25–2.00)	0.52
Ki67	>3%	0.98 (0.68–1.41)	0.91
Systemic therapy		0.73 (0.36–1.48)	0.38
Provider	A (comparison)	-	-
	B	1.09 (0.63–1.90)	0.76
	C	1.45 (0.79–2.65)	0.23

## Data Availability

Data will be available upon reasonable request to the authors.
